# The lysine acetyltransferase GCN5 contributes to human papillomavirus oncoprotein E7‐induced cell proliferation via up‐regulating E2F1

**DOI:** 10.1111/jcmm.13806

**Published:** 2018-08-06

**Authors:** Lijun Qiao, Qishu Zhang, Weifang Zhang, Jason J. Chen

**Affiliations:** ^1^ The Cancer Research Center and Department of Microbiology School of Basic Medical Sciences Shandong University Jinan Shandong China; ^2^ Department of Microbiology and Key Laboratory of Infection and Immunity of Shandong Province School of Basic Medical Sciences Shandong University Jinan Shandong China

**Keywords:** Cdk1, E2F1, G1 checkpoint, GCN5, human papillomavirus

## Abstract

General control nondepressible 5 (GCN5), the first identified transcription‐related lysine acetyltransferase (KAT), is an important catalytic component of a transcriptional regulatory SAGA (Spt‐Ada‐GCN5‐Acetyltransferase) and ATAC (ADA2A‐containing) complex. While GCN5 has been implicated in cancer development, its role in cervical cancer is not known. The human papillomavirus (HPV) oncoprotein E7 abrogates the G1 cell cycle checkpoint and induces genomic instability, which plays a central role in cervical carcinogenesis. In this study, we observed that GCN5 was up‐regulated in HPV E7‐expressing cells, knockdown of GCN5 inhibited cell cycle progression and DNA synthesis in HPV E7‐expressing cells. Notably, GCN5 knockdown reduced the steady‐state levels of transcription factor E2F1. Depletion of E2F1 caused G1 arrest while overexpression of E2F1 rescued the inhibitory effects of GCN5 knockdown on G1/S progression in HPV E7‐expressing cells. Results from chromatin immunoprecipitation (ChIP) assays demonstrated that GCN5 bound to the E2F1 promoter and increased the extent of histone acetylation within these regions. GCN5 also acetylated c‐Myc and increased its ability to bind to the E2F1 promoter. Knockdown of c‐Myc reduced the steady‐state levels of E2F1 and caused G1 arrest. These results revealed a novel mechanism of E7 function whereby elevated GCN5 acetylates histones and c‐Myc to regulate E2F1 expression and cell cycle progression.

## 1 INTRODUCTION

As one of the most common malignancies in women worldwide,^1^ cervical cancer is commonly associated with infection of the high‐risk human papillomaviruses (HR‐HPV).^2^, ^3^ HPVs are small DNA viruses that replicate in squamous epithelial cells. HPV oncogenic proteins E6 and E7 can bind and degrade tumour suppressors p53 and the retinoblastoma (pRb) protein family, respectively, thus regulate key cellular processes such as proliferation and transformation.^4^, ^5^ E7 from the HR‐HPV types (such as HPV‐16 and HPV‐18) can abrogate cell cycle checkpoints and induce genomic instability.^5^ Although many studies on E7 regulating cell cycle have been conducted, the mechanism is still not fully understood.

Cell cycle progression is regulated by cyclins and cyclin‐dependent kinases (Cdks) at several checkpoints, including the G1, G2/M, spindle and postmitotic G1 checkpoints.^6^, ^7^ The G1 checkpoint is the key checkpoint to determine whether cells enter the S phase and proliferate. In normal case, cells arrest in G1 phase with DNA damage, which protects cells with damaged DNA from being replicating and allows the cellular repair systems to fix the damaged DNA. HPV‐16 E7 abrogates the G1 cell cycle checkpoint in part by degrading pRb,^6^ resulting in the E2F transcription factor family member dissociation from pRb. Free E2Fs then bind to in the promoter region of a number of genes whose products are involved in cell cycle regulation or in DNA replication.^8^

Acetylation, especially histone acetylation, plays a role in regulating gene expression.^9^ Alteration of histone 3 and 4 acetylation level has been observed in a variety of cancers, including cervical cancer.^10^, ^11^ Histone deacetylase 1 (HDAC1) and HDAC2 are up‐regulated in cervical dysplasia and invasive cervical carcinoma.^12^ In cervical cancer HeLa cells, down‐regulation of HDAC2 expression inhibited cell proliferation and induced cell cycle arrest.^10^ HPV E7 has been reported to modulate histone acetylation through several mechanisms. E7 was shown to associate with p300/CBP‐associated factor (pCAF) and reduce its ability to acetylate histones.^13^ Moreover, HPV E7 facilitated DNA replication by activating E2F2 transcription through its interaction with HDACs and inhibiting HDAC binding to the E2F2 promoter.^14^, ^15^ E7 proteins were found to bind to hypoxia‐inducible factor 1 (HIF‐1α) and enhance its transcription activities by inhibiting binding of HDACs.^16^ E7 could directly target cdc25A transcription and maintains cdc25A gene expression by disrupting Rb/E2F/HDAC‐1 repressor complexes during deregulation of cell cycle arrest.^17^ Conversely, HPV E7 recruited HDACs to the promoter of interferon response factor 1 (IRF1) and suppressed its transcriptional activity.^18^, ^19^

General control nondepressible 5 (GCN5), the first identified transcription‐related lysine acetyltransferase (KAT), is an important catalytic component of a transcriptional regulatory complex.^20^, ^21^ GCN5 plays a key role in a broad range of cellular functions, including cell proliferation, differentiation, telomere maintenance and DNA damage repair.^22^, ^23^, ^24^, ^25^ As a key catalytic component of the larger SAGA and ATAC complexes, GCN5 preferentially acetylates lysines 9, 14, 27 and 56 of histone H3 and lysines 8 and 16 of H4.^20^, ^21^, ^26^, ^27^ In recent years, GCN5 has also been implicated a function in certain oncogenic processes. In breast cancer cells, HBXIP (hepatitis B X‐interacting protein) oncoprotein promotes the migration of breast cancer cells through modulating microtubule acetylation mediated by GCN5.^28^ GCN5 is required for invariant natural killer T (iNKT)‐cell development through EGR2 acetylation.^29^ GCN5 positively regulates T‐cell activation and loss of GCN5 functions impaired T‐cell proliferation.^30^ GCN5 promotes human hepatocellular carcinoma progression by enhancing de novo transcription of the AIB1 gene.^31^ GCN5 increases the stability of c‐Myc, one of the most frequently overexpressed genes in human cancer,^32^, ^33^ by acetylating its K323 residue.^34^ Moreover, GCN5 directly interacts with E2F1 and acetylates H3K9 on its promoter region to facilitate the expression of Cyclin E and Cyclin D1 to promote lung cancer cell proliferation and tumour growth.^22^ The role of GCN5 in cervical cancer has not been reported yet.

In this study, we investigated the role of GCN5 in cell proliferation of HPV‐16 E7‐expressing cells and explored the mechanism by which GCN5 performs its functions. GCN5 was found to be up‐regulated in HPV‐16 E7‐expressing cells. Down‐regulation of the GCN5 reduced E7‐induced G1 checkpoint abrogation. GCN5 mediated cell cycle proliferation by regulating E2F1 expression that was associated with increased acetylating histone 3 lysine 9 (H3K9) in the promoter of E2F1, overexpression of E2F1 rescued the inhibitory effect of GCN5 knockdown on G1/S transition. We also found that GCN5 regulated E2F1 expression by acetylating and stabilizing c‐Myc, which bond and regulated E2F1 and promoted cell cycle proliferation.

## 2 MATERIALS AND METHODS

### 2.1 Cell culture

Spontaneously immortalized human foreskin keratinocytes (NIKS cells) were described previously^35^ and cultured on mitomycin C‐treated J2‐3T3 feeder cells with E medium composed Ham's F12 medium and Dulbecco's modified Eagle medium (DMEM) (3:1) plus 5% foetal bovine serum (FBS). Cells of the human telomerase reverse transcriptase‐expressing human retinal pigment epithelium cell line RPE1 were maintained in Ham's F‐12‐DMEM medium (1:1) plus 10% FBS.

NIKS cells expressing HPV‐16 E7 and RPE1 cells expressing HPV‐16 E7 were established using a pBabe retroviral system as described previously.^36^ NIKS cells and RPE1‐derived cell lines were maintained in puromycin and used within 15 passages, and the expression of E7 protein was confirmed by Western blot after cultured for every 5 passages. All cells were cultured in medium with the addition of penicillin and streptomycin at 37°C with 5% CO_2_.

### 2.2 RNA extraction and reverse transcription (RT)‐PCR

The extraction of RNA was carried out using the TRIzol Reagent (Invitrogen) according to the manufacturer's instructions. cDNA was synthesized using random primers with the PrimeScript™ RT Reagent Kit with gDNA Eraser (Takara) according to manufacturer's instructions. Amplification of PCR products was quantified using SYBR^®^ Premix Ex Taq™ (Takara) and monitored on a DNA Engine Peltier thermal cycler (Bio‐Rad) equipped with a Chromo4 Real‐Time PCR detection system (Bio‐Rad). The following cycling conditions were used: initial denaturation at 95°C for 3 minutes, followed by 40 cycles of 95°C for 15 seconds, 60°C for 20 seconds and 72°C for 30 seconds. The PCR primers were as follows: GCN5 forward, 5′‐GCAAGGCCAATGAAACCTGTA‐3′; GCN5 reverse, 5′‐TCCAAGTGGGATACGTGGTCA‐3′; E2F1 forward, 5′‐CATCCCAGGAGGTCACTTCTG‐3′; E2F1 reverse, 5′‐GACAACAGCGGTTCTTGCTC‐3′; GAPDH forward, 5′‐GCACCGTCAAGGCTGAGAAC‐3′; GAPDH reverse, 5′‐TGGTGAAGACGCCAGTGGA‐3′.

Expression levels were assessed in triplicate and normalized to GAPDH levels, and graphs represent the combined results for three independent biological replicates.

### 2.3 siRNA and transfection

Cells were seeded in 6‐cm dishes at 3 × 10^4^ cells and cultured in medium without antibiotics for 24 hour. Chemically modified Stealth small interfering RNA (siRNA) targeting GCN5, E2F1, c‐Myc and control siRNA were purchased from Guangzhou RiboBio (RiboBio, Guangzhou, China) and transfected into cells using Lipofectamine 2000 (Invitrogen, Life Technologies, CA, USA) according to manufacturer's instructions. Cells were transfected with siRNA at a concentration of 20 nmol/L. The sequences of siRNAs are as follows: siGCN5‐1 5′‐GGAAAUGCAUCCUGCAGAU‐3′; siGCN5‐2 5′‐GAGGCCUCAUUGACAAGUA‐3′; siE2F1‐1 5′‐GUCACGCUAUGAGACCUCA‐3′; siE2F1‐2 5′‐GGACCUUCGUAGCAUUGCA‐3′; sic‐Myc‐1 5′‐CGUCCAAGCAGAGGAGCAA‐3′; sic‐Myc‐2 5′‐CCAAGGUAGUUAUCCUUAA‐3′.

Thirty‐six hours after transfection, the cells were treated with DMSO or 10 μg/mL bleomycin and incubated for an additional 36 hours. Cells were harvested for protein knockdown analysis by Western blotting or for cell cycle analysis by flow cytometry.

### 2.4 Plasmids and transfection

The plasmid pcDNA3.0‐HA‐c‐Myc encodes c‐Myc with a HA tag at the C‐terminus in vector pcDNA 3.0. The plasmid pCMV‐E2F1 (Addgene) encodes human E2F1 with the C‐terminal HA epitope tag. To construct c‐Myc variants (K323R, K323Q), mutagenesis was performed using the QuikChange site‐directed mutagenesis kit and QuikChange XL site‐directed mutagenesis kit (Stratagene). Mutations were confirmed by DNA sequencing. Transient transfection was performed with Lipofectamine 2000 (Invitrogen, Life Technologies, CA, USA).

### 2.5 Flow cytometry

For cell cycle experiment, cultured cells were treated with phosphate‐buffered saline (PBS) or bleomycin (Alexis Biochemicals) (10 μg/mL in PBS). At 36 hours later, cells were fixed in 70% ethanol, treated with 50 μg/mL RNase A plus 50 μg/mL propidium iodide (PI). The PI‐stained cells were analysed by flow cytometry. Cell cycle analysis was performed using FlowJo software (Becton Dickinson).

For the bromodeoxyuridine (BrdU) labelling experiment, BrdU (final concentration, 20 μmol/L) was added to the medium 2 hours before collection of cells. After fixation, cells were permeabilized with 2 N HCl‐0.5% Triton X‐100, neutralized with 0.1 mol/L sodium tetraborate, stained with monoclonal anti‐BrdU (BD Biosciences) followed by treatment with antimouse IgG F(ab)2‐fluorescein isothiocyanate (FITC) (Sigma) and counterstained with PBS‐7‐aminoactinomycin D (7‐AAD)‐RNase A. Immunofluorescent cells were analysed by use of FACSCalibur (BD) and FCSexpress.

### 2.6 Western blot

Total cellular proteins were extracted with radioimmunoprecipitation assay (RIPA) lysis buffer, and a Western blot assay was performed with specific antibodies against GCN5 (sc‐55559, Santa Cruz), Cdk1 (610038, BD Biosciences), Cdk4 (sc‐260, Santa Cruz), E2F1(sc‐251, Santa Cruz), Cyclin A (sc‐751, Santa Cruz), pRb (554136, BD Biosciences), p53 (sc‐98, Santa Cruz) and Tubulin (T‐4026, Sigma), HPV‐16 E7 (sc‐6981, Santa Cruz,), c‐Myc (ab56, Abcam), acetylated‐H3K9 (ab177177, Abcam), histone 3 (3638P, CST), acetylated‐lysine (9441S, CST), IRDye 800CW goat antimouse IgG (LI‐COR, 926‐32210), IRDye 800CW goat anti‐rabbit IgG (LI‐COR, 926‐32211). The bands of protein were detected using an Odyssey infrared imaging system (LI‐COR, Lincoln, NE) and quantified using ImageJ (NIH).

### 2.7 ChIP assay

The chromatin immunoprecipitation (ChIP) assay was performed using a ChIP assay kit from Millipore, following the manufacturers’ protocol. Immunoprecipitations were performed using anti‐GCN5, anti‐c‐Myc, anti‐HA or control IgG antibodies. PCR was performed with the primers designed from the sequences of the human E2F1 gene as follows: forward 5′‐AAGCCAATAGGAACCGCCG‐3′, reverse 5′‐AGTCCCGGCCACTTTTACG‐3′ (for GCN5); forward 5′‐TGAGGATGGAAGAGGTGGCT‐3′; reverse 5′‐TTCTGCACGTGACCCTCAAC‐3′ (for c‐Myc or HA).

### 2.8 Statistical analysis

Data are presented as means and standard deviations (SDs). The differences between means were evaluated using Student *t* test. *P* values of ≤ 0.05 were considered significant.

## 3 RESULTS

### 3.1 GCN5 expression was up‐regulated in HPV‐16 E7‐expressing cells

**Figure 1:**
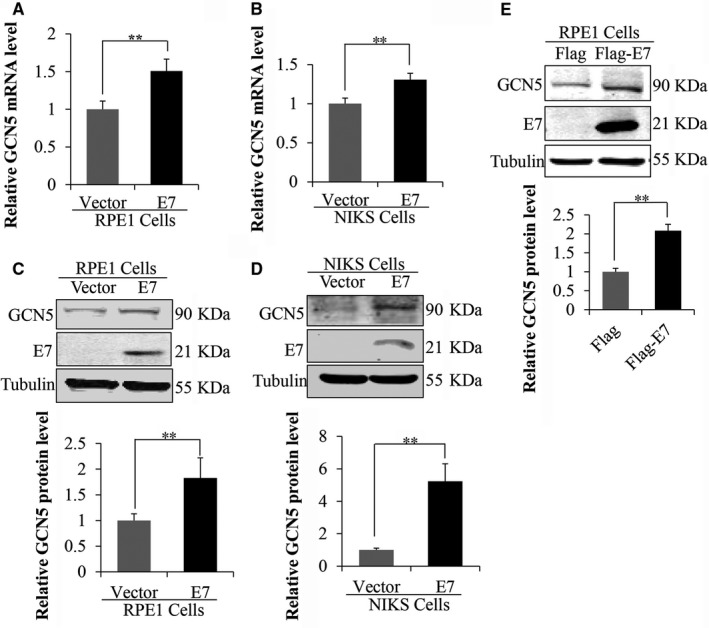
GCN5 expression was up‐regulated in HPV‐16 E7‐expressing cells. (A) and (B) GCN5 mRNA levels in NIKS and RPE1 cells determined by real‐time PCR. (C) and (D) Expression of GCN5 and HPV‐16 E7 proteins in NIKS and RPE1 cells. The steady‐state levels of GCN5 and E7 proteins in NIKS and RPE1 cells determined by Western blot. (E) The protein level of GCN5 was measured by Western blot after transfected with plasmids encoding HPV‐16 E7 for 48 h. Data from a representative experiment of 3 are shown, *P < 0.05; **P < 0.01

E7 oncogene plays a key role in cervical carcinogenesis and abrogates the G1 checkpoint.^6^ Our recent study showed that HPV‐16 E7 abrogated the G1 checkpoint by up‐regulating the Cdk1, Cdc6, WDHD1 and cancerous inhibitor of protein phosphatase 2A (CIP2A),^37^, ^38^, ^39^, ^40^ more detailed mechanisms remain to be elucidated. Overexpression of GCN5 promotes cell growth and the G1/S phase transition.^22^ We therefore speculated that GCN5 may play a role in E7‐mediated cell cycle control. To test this, we firstly used HPV‐16 E7‐expressing NIKS cells (NIKS‐E7).^41^ NIKS cells exhibit many characteristics of early‐passage human keratinocytes including stratification, differentiation and the ability to sustain the HPV life cycle^42^, ^43^ and grow relatively well in culture. We found that the GCN5 mRNA level was up‐regulated (~1.4‐fold) in E7‐expressing NIKS cells (Figure 1A). As keratinocytes are difficult to achieve high transfection efficiencies in our experimental conditions, we also used RPE1 cells to express HPV‐16 E7 (RPE1‐E7). The RPE1 cells have been used in our recent HPV‐related functional studies.^35^, ^36^, ^39^ Similar to what was observed in keratinocytes, GCN5 mRNA levels were increased (~1.5‐fold) in E7‐expressing RPE1 cells (Figure 1B). Next, we examined the steady‐state level of GCN5 protein in E7‐expressing cells. As shown in Figure 1C,D, the levels of GCN5 protein were significantly up‐regulated in both RPE1‐E7 cells (~1.8‐fold) and NIKS‐E7 cells (~5‐fold). To directly demonstrate the ability of E7 to up‐regulate GCN5, we transfected cells with plasmids encoding HPV‐16 E7 and detected the expression of GCN5. As shown in Figure 1E, the steady‐state level of GCN5 protein was increased upon E7 transfection. These results demonstrate that GCN5 expression was up‐regulated in HPV‐16 E7‐expressing cells.

### 3.2 GCN5 siRNA knockdown caused G1 arrest and inhibited DNA synthesis in HPV‐16 E7‐expressing cells

**Figure 2:**
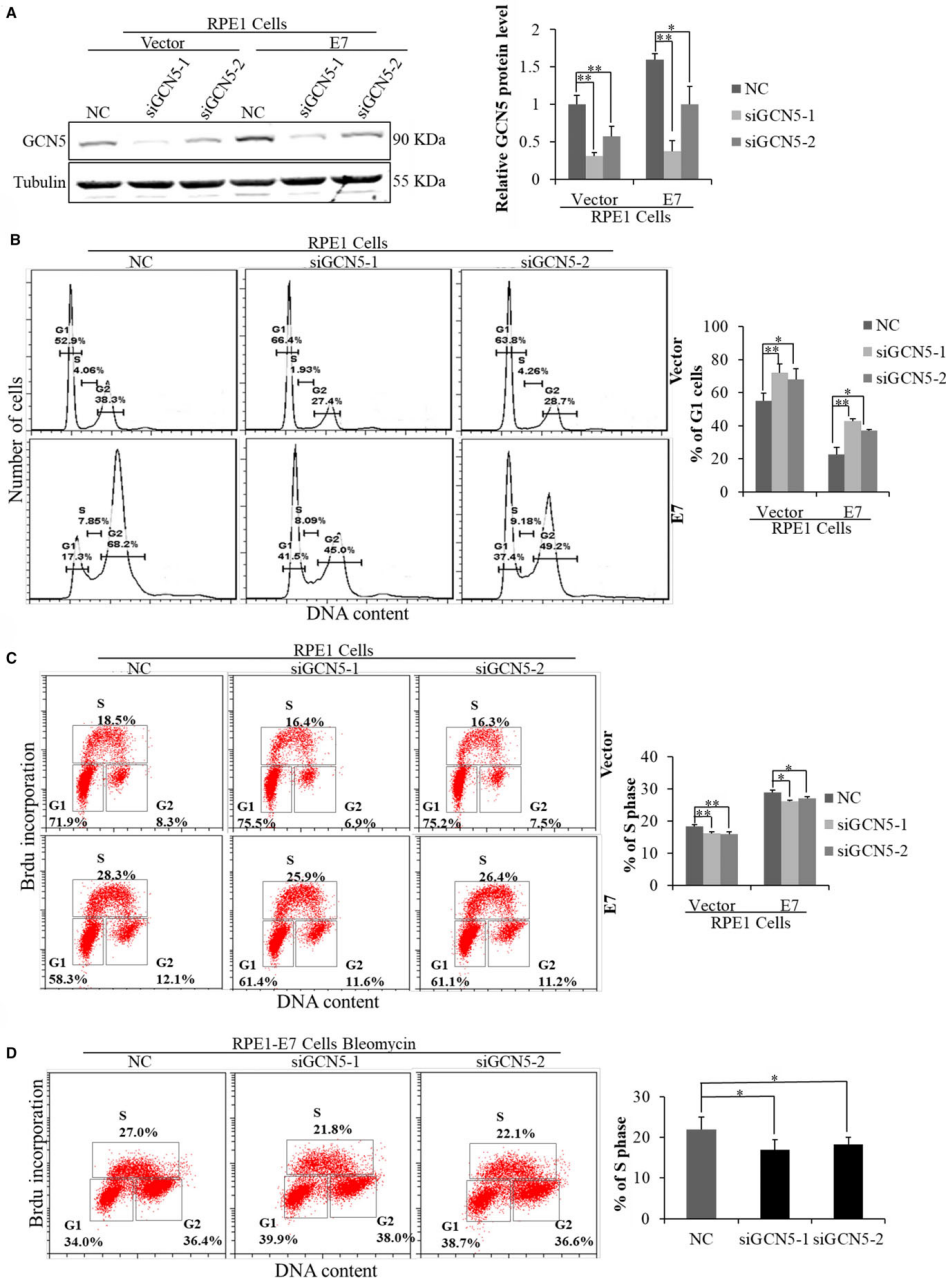
GCN5 knockdown caused G1 arrest and inhibited DNA synthesis in HPV‐16 E7‐expressing cells. (A) The protein level of GCN5 was measured by Western blot after transfected with siRNAs targeting GCN5 for 48 h. (B) Flow cytometry of cells treated with bleomycin for 36 h after transfected cells with GCN5 siRNA for 24 h and then stained with PI. G1, S and G2 phases are indicated and quantified. (C) Flow cytometry of cells transfected with GCN5 siRNA for 48 h and labelled with 20 nmol/L BrdU for 2 additional hours. Cells were stained with anti‐BrdU antibody, counterstained with 7‐AAD and analysed by flow cytometry. (D) Flow cytometry of cells treated with bleomycin for 36 h after transfected cells with GCN5 siRNA for 24 h. Data from a representative experiment of 3 are shown, *P < 0.05; **P < 0.01

To test the potential role of GCN5 in E7‐mediated cell cycle control, we used two independent siRNAs. The steady‐state level of GCN5 protein was down‐regulated (to 0.2‐fold with siGCN5‐1, to 0.5‐fold with siGCN5‐2) after transfection with siRNAs targeting GCN5 in RPE1‐E7 cells (Figure 2A). Next, we examined the effect of GCN5 knockdown on cell cycle profiles in E7‐expressing and vector‐containing RPE1 cells. No significant effects on cell cycle profile were observed when regularly cultured RPE1 cells containing the vector or expressing E7 were treated with GCN5 siRNAs (data not shown). To explore the role of GCN5 in G1 checkpoint, we treated cells with bleomycin (10 μg/mL), which causes both single‐ and double‐strand DNA damage and induces normal cells to arrest at the G1 phase while cells expressing HPV E7 go through S phase and arrest at G2 phase, as we showed previously.^37^ Consistent with what we have observed, upon treatment with bleomycin, fewer cells (17.3% vs 52.9%) arrested at the G1 phase in E7‐expressing cells than in the vector control cells (Figure 2B), indicating abrogation of the G1 checkpoint by HPV E7. Notably, knockdown of GCN5 led to an increase in the G1 peak from 17.3% to 41.5% (by siGCN5‐1) and 37.4% (by siGCN5‐2) in E7‐expressing cells (Figure 2B). Abrogation of the G1 checkpoint indicates that DNA replication occurs in the presence of DNA damage by bleomycin. To demonstrate the role of GCN5 in promoting S phase entry, we transfected siRNAs targeting GCN5 into E7‐expressing cells and measured BrdU incorporation. Significantly, knockdown of GCN5 by siRNAs led to a mild by statistically significant reduction in BrdU incorporation in RPE1‐E7 cells (from 27.0% to 21.8% by siGCN5‐1 and 22.1% by siGCN5‐2) in bleomycin‐treated RPE1‐E7 cells (Figure 2C). These results demonstrate an important role of GCN5 in the G1 cell cycle control and S phase entry of E7‐expressing cells.

### 3.3 E2F1‐dependent regulation of Cdk1 by GCN5 in E7‐expressing cells

**Figure 3:**
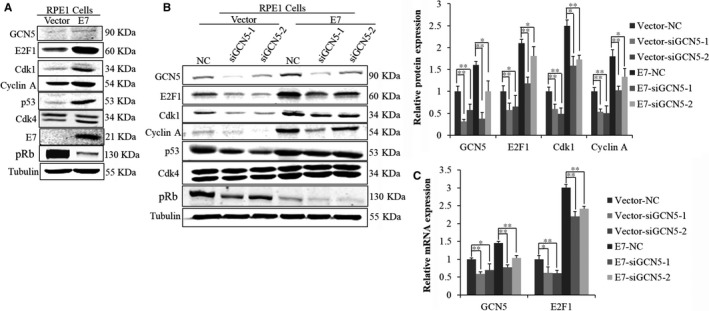
Knockdown of GCN5 by siRNAs decreased the expression of E2F1 and cell cycle‐related proteins. (A) Western blot analysis of cell cycle‐related proteins between Vector and E7. (B) Western blot analysis of cell cycle‐related proteins after transfection with GCN5 siRNAs. (C) GCN5 and E2F1 mRNA levels in RPE1 cells were determined by real‐time PCR after transfection with GCN5 siRNAs. Data from a representative experiment of 3 are shown, *P < 0.05; **P < 0.01

The progression of cell cycle is regulated by Cdks and cyclins at several checkpoints. Cdk2 is a major player for S phase entry, but its function can be compensated by Cdk1 in its absence,^44^ as we demonstrated in E7‐expressing cells.^37^, ^39^ To explore the mechanism by which GCN5 regulates cell cycle, we detected the expression of genes involved in the G1 checkpoint, E2F1, Cdk1, Cyclin A, p53, pRb and Cdk4. The protein levels of E2F1, Cdk1, Cyclin A, p53 were up‐regulated in E7‐expressing cells (Figure 3A).

**Figure 4:**
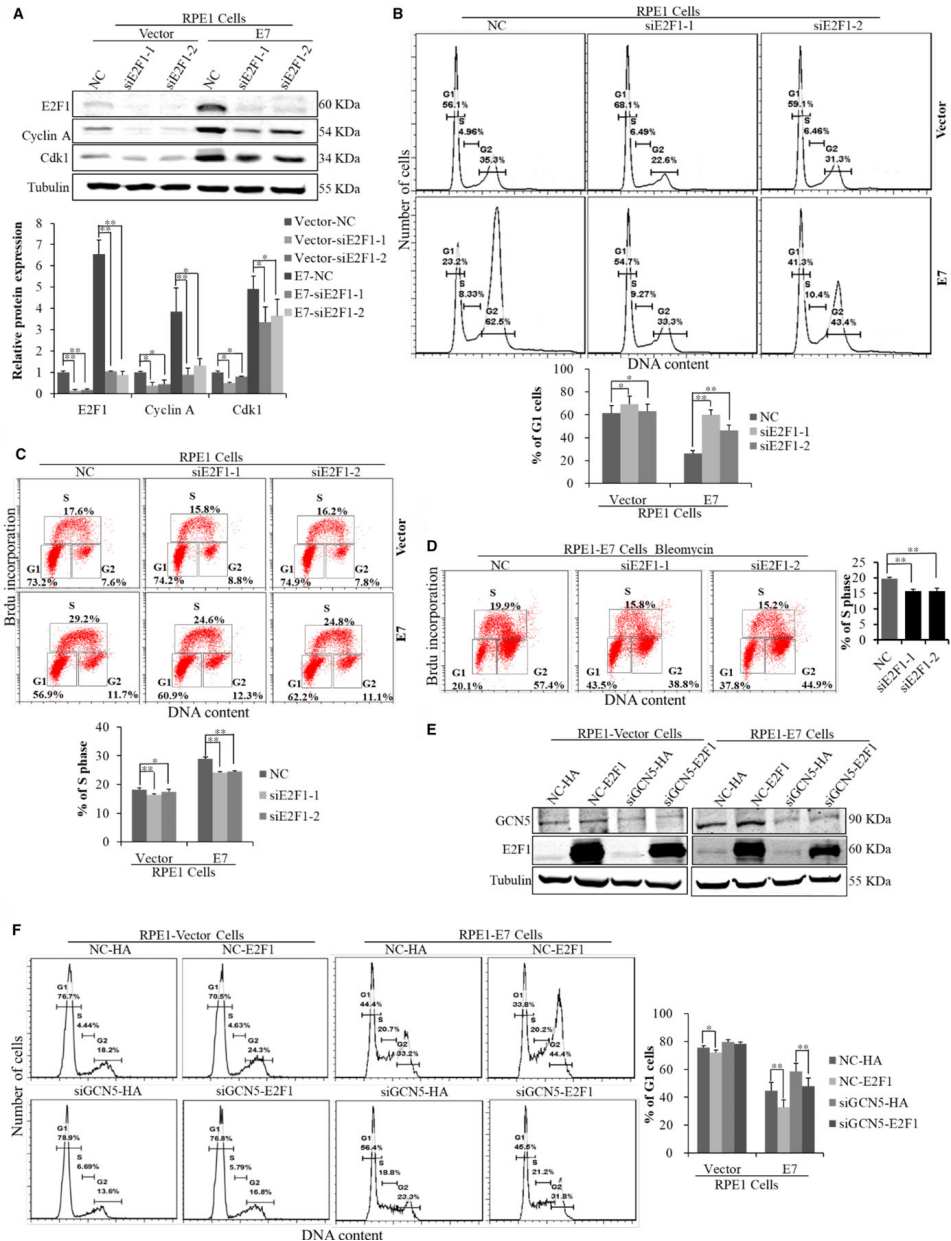
E2F1‐depended regulation of Cdk1 by GCN5 in E7‐expressing cells. (A) Western blot analysis of cell cycle‐related proteins after transfection with GCN5 siRNAs. (B) Flow cytometry of cells treated with bleomycin for 36 h after transfected cells with E2F1 siRNA for 24 h and then stained with PI. (C) Flow cytometry of cells transfected with E2F1 siRNA for 48 h and labelled with 20 nmol/L BrdU for 2 additional hours. Cells were stained with anti‐BrdU antibody, counterstained with 7‐AAD and analysed by flow cytometry. (D) Flow cytometry of cells treated with bleomycin for 36 h after transfected cells with E2F1 siRNA for 24 h and labelled with 20 nmol/L BrdU for 2 additional hours. (E) Western blot analysis of E2F1 after cells was transfected with pCMV or pCMV‐E2F1 plasmid. (F) Flow cytometry of cells treated with bleomycin for 36 h after transfection with GCN5 siRNA, and pCMV or pCMV‐E2F1 plasmid, and then stained with PI. Data from a representative experiment of 3 are shown, *P < 0.05; **P < 0.01

E2F1 has been generally considered as a positive regulator of transcription.^45^ E2F1 binds in the upstream regions of the human Cdk1 and Cyclin A genes and accelerates their transcriptions.^46^, ^47^ We transfected cells with siRNAs specific to GCN5 and found the protein levels of E2F1, Cdk1, Cyclin A and pRb were down‐regulated (Figure 3B). Furthermore, the mRNA level of E2F1 was also down‐regulated with GCN5 knockdown (Figure 3C). As GCN5 positively regulated E2F1,^22^ we believe it may do so as well in E7‐expressing cells to control cell cycle. To test this, we transfected cells with siE2F1s and examined the knockdown efficiency and detected the expression of Cdk1 and Cyclin A. As expected, the steady‐state levels of Cdk1 and Cyclin A proteins were decreased (Figure 4A), an observation consistent with GCN5 knockdown, suggesting that E2F1 plays a role in GCN5 down‐regulation resulted Cdk1 and Cyclin A decrease. Next, we examined the ability of E2F1 to modulate the G1 checkpoint in E7‐expressing and vector‐containing RPE1 cells after E2F1 knockdown by treating cells with bleomycin. Notably, knockdown of E2F1 led to an increase in the G1 peak (siE2F1‐1, from 23.2% to 54.7%; siE2F1‐2, from 23.2% to 41.3%) in E7‐expressing cells (Figure 4B) and decrease in S phase (Figure 4C,D). To test whether down‐regulation Cdk1 and induction of G1 arrest by GCN5 knockdown was dependent on E2F1, we transfected cells with plasmid overexpressed E2F1. The expression of E2F1 was confirmed after transfection of E2F1 plasmid (Figure 4E). The expression of Cdk1 and Cyclin A increased significantly with overexpression of E2F1 (data not shown). Overexpression of E2F1 rendered E7‐expressing cells partially overcame G1 arrest after GCN5 knockdown (44.4% vs 33.8%). Knockdown GCN5 caused an accumulation of cells in G1 peak (44.4% vs 56.4%) while overexpression of E2F1 partly abrogated G1 arrest caused by GCN5 knockdown (56.4% vs 45.5%) in E7‐expressing cells (Figure 4F). Thus, E2F1 overexpression rescued the inhibitory effect of GCN5 knockdown on G1 arrest and Cdk1 and Cyclin A expressions in E7‐expressing cells.

### 3.4 GCN5 bond to the E2F1 promoter and increased histone H3 acetylation in E7 ‐expressing cells

**Figure 5:**
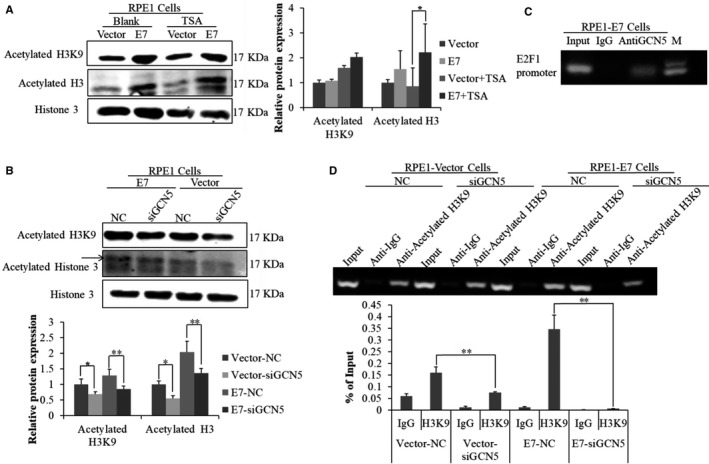
GCN5 bond to and increased the histone H3 acetylation of the E2F1 promoter in E7‐expressing cells. (A) The steady‐state of acetylated H3K9 and acetylated H3 was measured by Western blot after treating cells with TSA for 24 h. (B) The steady‐state level of acetylated H3K9 and acetylated H3 was measured by Western blot after transfected with siGCN5‐1 targeting GCN5 for 48 h. (C) ChIP assay was performed using GCN5 antibody in cultured RPE1‐E7 cells. The GCN5‐associated E2F1 promoter binding in the ChIP samples was detected by real‐time PCR with E2F1 promoter primers. (D) ChIP assay was performed using acetylated H3K9 antibody after transfected with siRNA targeting GCN5 for 48 h. Data from a representative experiment of 3 are shown, *P < 0.05; **P < 0.01

As GCN5 is a histone acetyltransferase, it may acetylate histones in the cells, and we therefore examined histone 3 lysine 9 (H3K9) acetylation in E7‐expressing cells. As shown in Figure 5A, the acetylated H3K9 was higher in E7‐expressing cells, especially after treatment with histone deacetylase inhibitor trichostatin A (TSA). The acetylation level of H3K9 was decreased when cells were treated with siGCN5 (Figure 5B), indicating that GCN5 was responsible for the acetylation level of H3K9 in E7‐expressing cells. As the expression of E2F1 was markedly regulated by GCN5 at both protein and mRNA levels in E7‐expressing cells (Figure 3B,C) and GCN5 was shown to bind E2F1 promoter in lung cancer cell lines,^22^ we believe that GCN5 binds E2F1 promoter in E7‐expressing cells. To test this possibility, chromatin immunoprecipitation (ChIP) was used to evaluate the association of GCN5 on the E2F1 promoter. As shown in (Figure 5C), the promoter region of E2F1 gene was significantly immunoprecipitated by the GCN5 antibody, indicating that endogenous GCN5 binds to the E2F1 promoters. To further establish the role of GCN5 in acetylating histones of E2F1 promoter, ChIP assay was performed to examine the acetylation level of H3K9 on chromatin by treating cells with siGCN5. As shown in Figure 5D, the acetylation level of H3K9 binding to the promoter of E2F1 was significantly decreased with GCN5 knockdown. These results indicate that GCN5 binds to and increases the histone H3 acetylation of the E2F1 promoter in E7‐expressing cells. These results indicate that GCN5 regulates overall H3K9 acetylation as well as those on the E2F1 promoter.

### 3.5 Acetylation of c‐Myc by GCN5 contributed to its stability and ability to regulate E2F1 expression in E7‐expressing cells

**Figure 6:**
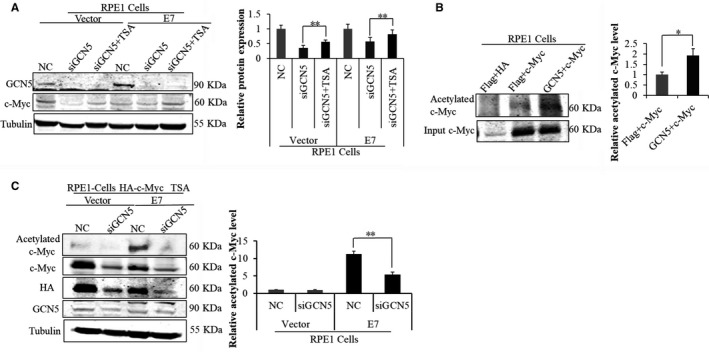
c‐Myc was acetylated and stabilized by GCN5 in E7‐expressing cells. (A) The steady‐state level of GCN5 and c‐Myc were measured by Western blot after treating cells with siGCN5‐1 for 24 h and additional 24 h for TSA treatment. (B) The level of acetylated c‐Myc and input c‐Myc was measured by Western blot after treating cells with plasmids Flag or Flag‐GCN5 and HA or HA‐c‐Myc for 48 h. (C) Western blot analysis of acetylated c‐Myc, c‐Myc, HA and GCN5 with GCN5 siRNA and TSA treatment. Data from a representative experiment of 3 are shown, *P < 0.05; **P < 0.01

c‐Myc and E2F1 can activate each other's transcription and form a positive feedback loop.^48^, ^49^ c‐Myc is up‐regulated in HPV‐positive cervical cancer.^50^ It was reported that the ability of c‐Myc to activate transcription partly relies on recruiting cofactor complexes including GCN5.^34^ The c‐Myc protein could be acetylated by GCN5/PCAF, resulting in an increase in protein stability.^34^ As the expression levels of both GCN5 and E2F1 were high in E7‐expressing cells (Figure 1), we speculated that c‐Myc could be acetylated by GCN5 in E7‐expressing cells to regulated E2F1 expression. To test this possibility, we examined the steady‐state levels of c‐Myc after GCN5 knockdown. For this, we treated E7‐expressing cells with siGCN5 and TSA and detected c‐Myc by Western blot. As shown in Figure 6A, the protein level of c‐Myc was decreased after GCN5 knockdown and increased with TSA treatment. Significantly, the extent of c‐Myc acetylation was increased when cells were transfected with the plasmid‐encoding GCN5 (Figure 6B). The acetylation level of c‐Myc was higher in E7‐expressing cells than that in the vector control cells (Figure 6C).

**Figure 7:**
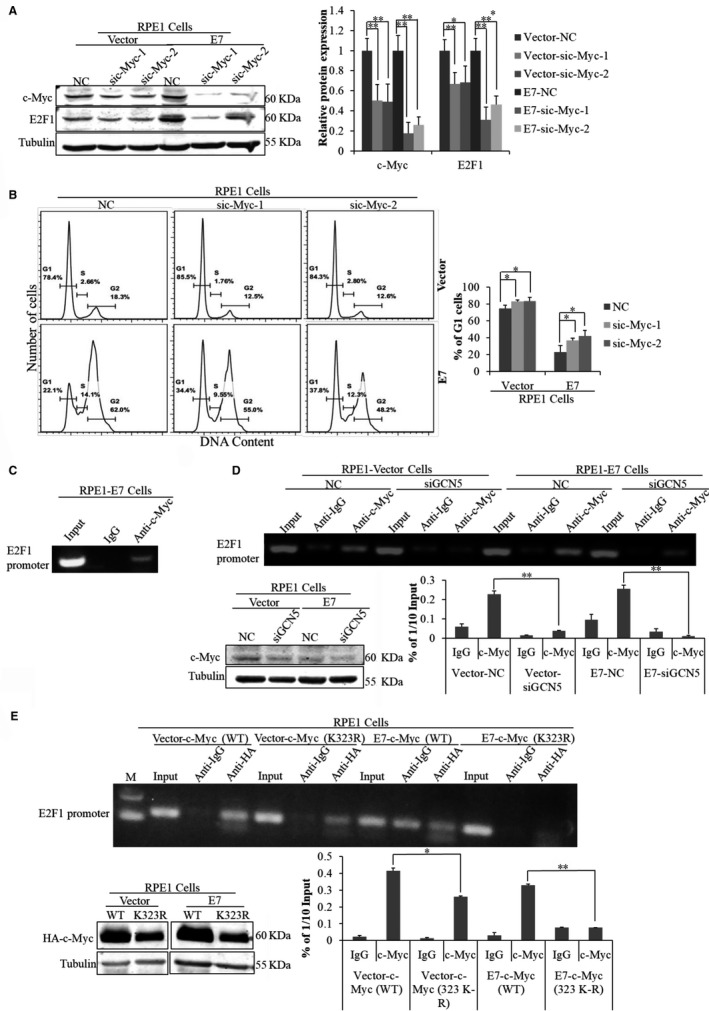
The level of c‐Myc binding to E2F1 promoter was affected by GCN5. (A) The steady‐state levels of E2F1 and c‐Myc were measured by Western blot after treating cells with sic‐Myc for 24 h and additional 24 h with TSA treatment. (B) Flow cytometry of cells treated with bleomycin for 36 h after transfection with c‐Myc siRNA for 24 h and stained with PI. (C) ChIP assay was performed using c‐Myc antibody in cultured RPE1‐E7 cells. (D) ChIP assay was performed using c‐Myc antibody after transfection with siRNA targeting GCN5 for 48 h and Western blot analysis of the steady‐state level of c‐Myc. (E) Western blot analysis of c‐Myc after treating cells with plasmids c‐Myc (K323R). ChIP assay was performed using HA antibody after treating cells with plasmids c‐Myc (K323R). WT, wild‐type. Data from a representative experiment of 3 are shown, *P < 0.05; **P < 0.01

Next, we examined the potential role of c‐Myc in E2F1 regulation and G1 checkpoint abrogation in HPV E7‐expressing cells. After transfection with sic‐Mycs, E2F1 expression was decreased (Figure 7A), suggesting a role of c‐Myc in E2F1 regulation. Notably, knockdown of c‐Myc in the presence of bleomycin led to an increase in the G1 peak (Figure 7B), indicating a role of c‐Myc in G1 checkpoint regulation in E7‐expressing cells. c‐Myc was shown to bind to the promoter of E2F1 and activated E2F1 transcription in normal cells.^51^ To confirm this in E7‐expressing cells, we used ChIP assay. As shown in Figure 7C, the promoter region of E2F1 gene was significantly immunoprecipitated with the c‐Myc antibody. Significantly, the level of c‐Myc binding to E2F1 promoters was significantly decreased when cells were treated with GCN5 siRNA (Figure 7D), suggesting that acetylation of c‐Myc by GCN5 affect the ability of c‐Myc binding to E2F1 promoters.

It was reported that GCN5 increases the stability and enhances transcription activation of c‐Myc by acetylating its K323 residue.^34^, ^52^ To test the possibility that K323 residue of c‐Myc is important for acetylation and stability, we constructed c‐Myc mutants with mutations at K323 (K323R, imitating the deacetylation status; K323Q, imitating the acetylation status). Consistent with previous observation that K323 acetylation affects c‐Myc stability, the steady‐state levels of K323R mutant of c‐Myc were lower than that of the wild‐type (Figure 7E). What is important, the relative level of c‐Myc binding to E2F1 promoters was also decreased for K323R (Figure 7E). However, the level of c‐Myc binding to E2F1 promoters was not increased when transfected with plasmids encoding c‐Myc (K323Q) that imitated the acetylation status of c‐Myc (data not shown). It was reported that K323 overacetylation might cause reduced c‐Myc protein stability while preserving stability in normal conditions.^53^ This observation may explain why c‐Myc was not always highly expressed in E7‐expressing cells.^40^ These results indicate that GCN5 could acetylate c‐Myc, stabilize it, affect its level of binding to the E2F1 promoter, regulate E2F1 expression and modulate cell cycle progression.

## 4 DISCUSSION

In this study, we investigated the mechanism underlying HPV‐16 E7 abrogation of the G1/S cell cycle checkpoint. GCN5 was found to be up‐regulated in HPV‐16 E7‐expressing cells. Down‐regulation of GCN5 reduced E7‐induced G1 checkpoint abrogation and the expression of several cell cycle‐related proteins. GCN5 modulated cell cycle progression by regulating E2F1 expression through H3K9 acetylating in the E2F1 promoter, and overexpression of E2F1 rescued the inhibitory effect of GCN5 knockdown on G1/S transition. We also found that acetylation of c‐Myc by GCN5 may contribute to E2F1 expression. Taken together, this study uncovered a novel function of GCN5 in high‐risk HPV E7‐mediated cell proliferation and has important implications in HPV‐associated cancers.

GCN5 is a histone acetyltransferase (HAT) that plays key roles in a broad range of cellular functions, including transcription,^54^ cell proliferation,^22^ differentiation,^23^ telomere maintenance^24^ and DNA damage repair.^25^ GCN5 promotes transcription by acetylating lysines 9, 14, 27 and 56 of histone H3 and lysines 8 and 16 of histone H4.^20^, ^21^, ^26^, ^27^ GCN5 promoted bone marrow‐derived mesenchymal stem cells (BMSCs)‐mediated angiogenesis by elevating H3K9ac levels on the promoter of VEGF.^55^ Overexpression of GCN5 promoted the growth of multiple lung cancer cell lines and facilitated the G1/S transition by acetylating histone 3 and histone 4 in the promoter of E2F1, Cyclin E1 and Cyclin D1.^22^ So far, there are no reports of GCN5 related to HPV and HPV‐associated cancers. In this study, we found that GCN5 was highly expressed in HPV E7‐expressing cells and plays a role in the progression of cell cycle in HPV E7‐expressing cells. We showed that in E7‐expressing cells, GCN5 regulated E2F1 expression that is accompanied by elevated level of histone 3 lysine 9 acetylation in the promoter of E2F1, is a novel observation for a potential role of GCN5 in HPV‐associated cancers.

E2F1, as a positive regulator of transcription, plays a crucial role in a variety of cellular functions, including cellular proliferation,^56^, ^57^ DNA repair,^58^ apoptosis^59^ and cell cycle.^60^ E2F1 up‐regulates genes required for cells to progress into late G1/S phase. E2F1 was found to bind in the upstream regions of the human Cdk1 and Cdk2 genes and regulate their transcription.^46^, ^61^ E2F1 also binds to the promoter of Cyclin A and regulated its expression, resulting in induction of S‐phase entry.^47^ E2F1 has been found to be deregulated in many types of cancers, including cervical cancer.^62^ E2F1 overexpression has been reported to promote cervical carcinogenesis.^63^ HPV E7 can bind and promote the degradation of retinoblastoma protein (pRb), resulting in the release of E2F1 from the pRb‐E2F complex and thereby promote malignant transformation of cervical epithelium.^64^, ^65^ pRb is an established target of E7, and E2F1 is an important regulator of Rb. However, E7‐mediated degradation of pRb is incomplete. GCN5 may represent a pRb‐independent mechanism of E7 action. There was also evidence showed that E7 bond to E2F1 and activated E2F1‐driven transcription in a pRb‐independent manner.^66^ E7 could also activate the transcription of E2F1 and increase its protein level by CIP2A.^40^, ^67^, ^68^ In our present study, we showed that the mRNA and protein levels of E2F1 were significantly up‐regulated in HPV E7‐expressing cells and down‐regulated when GCN5 was knocked down in E7‐expressing cells. These results demonstrated a positive association of GCN5 and E2F1 in E7‐expressing cells, suggesting that GCN5 controls G1/S transition by regulating E2F1 in E7‐expressing cells.

c‐Myc is one of the most commonly overexpressed genes in human cancers, including cervical cancer.^69^, ^70^ Acetylation of c‐Myc by CBP and GCN5/PCAF prevents its degradation and stimulation of c‐Myc transcriptional activity.^34^, ^71^ However, another study found that c‐Myc overacetylation at K323 may reduce its stability.^53^ c‐Myc plays a critical role for transformation of human cells by HPV E6 and E7.^72^, ^73^ c‐Myc interacts with and forms a specific complex with high‐risk type HPV E7 that augmented c‐Myc transactivation activity.^74^ The expression of c‐Myc correlated to HR‐HPV (including HPV‐16 and HPV‐18)‐infected epithelium compared with HR‐HPV‐negative epithelium.^75^ An enhanced c‐Myc level was also observed in cells expressing HPV E7.^76^, ^77^ However, some reports showed no significant difference in the expression of c‐Myc between vector and E7‐expressing cells.^78^ In this study, we did not find a significant increase in mRNA and protein level of c‐Myc in HPV E7‐expressing cells (data not shown), an observation consistent with our previous study.^40^ Although we found that the steady‐state levels of c‐Myc K323R mutant were lower than that of the wild‐type, the protein level of c‐Myc did not increase further upon transfection with plasmids encoding c‐Myc (K323Q) that imitated the acetylation status of c‐Myc (data not shown). These results implicate that the effect of c‐Myc acetylation on its stability is complicated. Nonetheless, acetylation of c‐Myc contributed to its binding to the E2F1 promoter. Further studies are needed to explore detailed role of c‐Myc acetylation by GCN5 to E2F1 regulation in HPV E7‐expressing cells. In summary, our data suggest a model where HPV E7 up‐regulates the expression of GCN5, which increases the expression of E2F1 through two pathways. In one case, GCN5 binds to the E2F1 promoter, increases histone H3 acetylation and promotes E2F1 transcription. In another case, GCN5 acetylates c‐Myc, which promotes E2F1 transcription. As an E2F1 target, Cdk1 involves in G1/S progression. This GCN5/c‐Myc/E2F1 pathway leads to abrogation of the G1 checkpoint in the presence of damaged DNA, genomic instability and contributing to HPV‐induced carcinogenesis (Figure S1).

## CONFLICT OF INTEREST

The authors confirm that there are no conflict of interests.
