# Supplementary figures and images for: The lysine acetyltransferase GCN5 contributes to human papillomavirus oncoprotein E7‐induced cell proliferation via up‐regulating E2F1
Source: J Cell Mol Med. 2018 Aug 6;22(11):5333–45. doi: 10.1111/jcmm.13806 (PMC6201343; doi:10.1111/jcmm.13806)

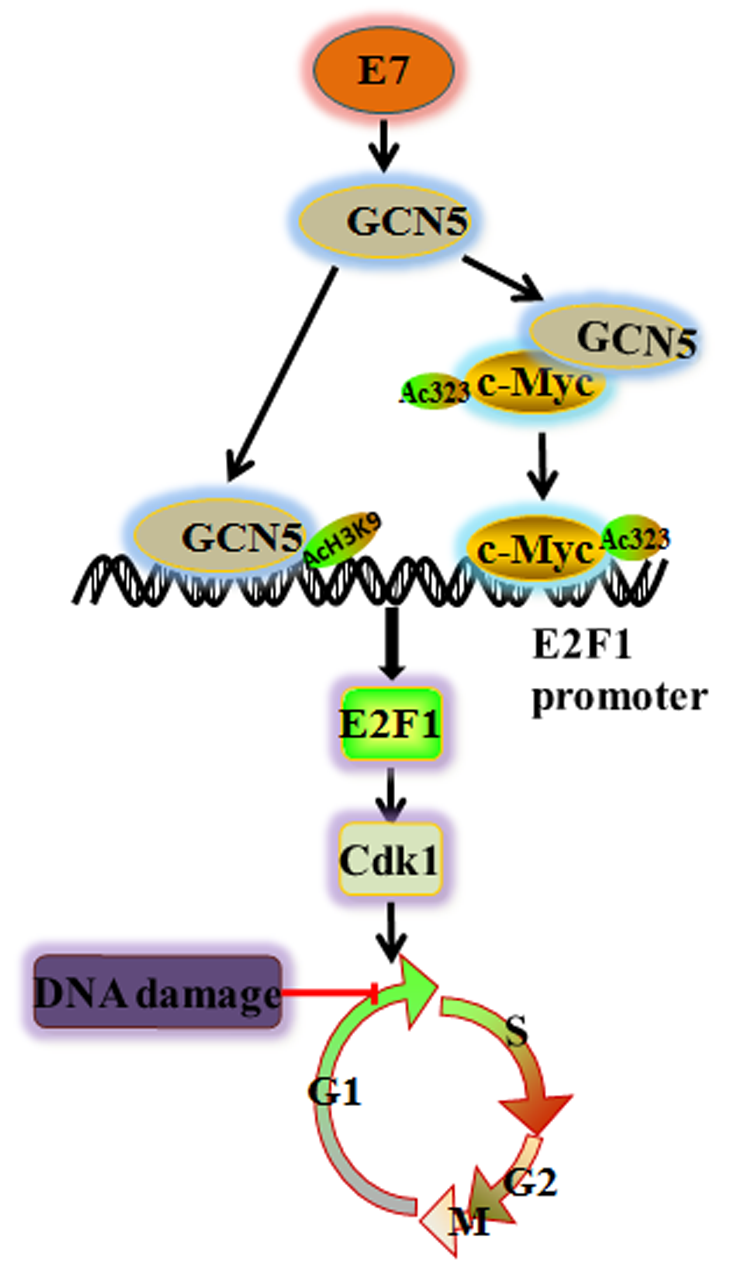

Supplement: Supplementary file 1 [file JCMM-22-5333-s001.tif]
